# Analysis of Autopsy Justifications by Medical Boards in Relation to the Manner and Cause of Death

**DOI:** 10.7759/cureus.83432

**Published:** 2025-05-03

**Authors:** Sanjay Kumar, Sawan Mundri, Kumar Shubhendu, Anand Kumar

**Affiliations:** 1 Department of Forensic Medicine and Toxicology, Rajendra Institute of Medical Sciences, Ranchi, IND

**Keywords:** autopsy, cause of death, custodial death, manner of death, medical board, natural death, unnatural death

## Abstract

Background

Autopsies serve a crucial role in elucidating ambiguous deaths, thereby providing legal clarity, wherein autopsy surgeons fulfill an indispensable function in ascertaining the manner of death, classified as natural, accidental, homicidal, suicidal, or indeterminate. Medical Board autopsies, which involve a collaborative multidisciplinary approach, yield a superior standard of medico-legal examination and are esteemed by judicial authorities. However, the extensive time and resources necessitated by these autopsies limit their application to atypical cases. There exists a salient requirement to rigorously assess the rationale behind Medical Board autopsies concerning the associated manner and causation of death.

Methodology

An observational analysis of a retrospective nature was performed at the Department of Forensic Medicine and Toxicology, Rajendra Institute of Medical Sciences, Ranchi, Jharkhand, India, assessing autopsy documentation between April 1, 2015 and March 31, 2020. A comprehensive review of 262 Medical Board autopsies was performed through consecutive sampling, evaluating post-mortem reports, police/magistrates' inquests, histopathological findings, biological samples, and toxicological analyses, among other relevant documents.

Results

Out of the 262 cases examined, 67.9% (178) of Medical Board autopsies were necessitated by custodial deaths, while 12.6% (33) arose from law enforcement exigencies. Natural causes led to 59.9% (157) of deaths, with unnatural causes contributing to 40.1% (105) of deaths. In categorizing unnatural deaths, it was noted that homicides were 49.5% (52), accidents represented 21.9% (23), suicides constituted 17.2% (18), and suspected medical negligence accounted for 7.6% (eight).

Conclusion

The outcomes underscore the necessity for transparent, standardized protocols governing the allocation of Medical Board autopsies, ensuring their deployment in circumstances demanding impartial, high-quality medico-legal evaluation, particularly in custodial deaths, allegations of malpractice, and cases anticipated to engage judicial scrutiny. Policymakers ought to emphasize resource distribution, establish objective criteria for Medical Board engagement, and formulate protocols to enhance both the efficacy and integrity of medico-legal investigations.

## Introduction

The allocation of justifications for performing autopsies by medical boards is a complex process influenced by various factors, including the need to accurately determine the manner and cause of death. Autopsies play a crucial role in forensic medicine, providing insights into the circumstances surrounding a death, especially when the cause is unclear or debatable. The decision to perform an autopsy is often guided by the potential to uncover critical information that can influence public health policies and legal outcomes.

Autopsies are often mandated in cases of unexplained or suspicious deaths to provide legal clarity. Autopsy surgeons play a limited but crucial role in determining the manner of death, which can be natural, accidental, homicidal, suicidal, or undetermined [1-2]. Autopsies also contribute to public health by identifying trends in mortality and potential public health threats. For instance, the Child Health and Mortality Prevention Surveillance Network highlights the importance of autopsies in identifying multiple causes of death in children, which can inform targeted interventions [3]. Autopsies also validate or refute clinical diagnoses, revealing discrepancies that can improve medical practices, with a study showing a significant rate of misdiagnosis, underscoring the value of autopsies in refining clinical assessments [4].

In cases involving multimorbidity, where a single underlying cause may not fully explain the death, a "multiple causes of death" approach is sometimes used to consider all conditions listed on the death certificate, providing a comprehensive view of mortality patterns [5-6]. Various statistical models, such as logistic regression, are often used to analyse factors influencing the decision to perform autopsies. These models consider variables like age, sex, ethnicity, and location of death, which can affect autopsy rates and outcomes [7]. The integration of forensic pathology with thorough investigations of death circumstances also helps to distinguish between "dying with" and "dying of" a condition, providing a clearer understanding of the cause of death [8].

Incorporating forensic findings into mortality registers can enhance the accuracy of mortality statistics. However, challenges such as under-reporting and misclassification of causes remain prevalent, particularly in cases involving external causes of death [9]. Despite their importance, autopsy rates are declining, especially among older populations and deaths occurring outside healthcare facilities. This trend limits the ability to obtain accurate mortality data, particularly for cardiovascular diseases [10]. Despite being invaluable for determining the manner and cause of death, autopsies face challenges such as potential biases in the selection of cases for autopsy.

The amalgamation of diverse causes-of-death data alongside forensic evidence into mortality statistics has the potential to significantly enhance the calibre of data accessible for both public health initiatives and legal applications. Nevertheless, it is imperative to undertake initiatives aimed at mitigating the obstacles associated with conducting autopsies and to bolster the precision of death certificates, thereby ensuring they accurately represent the intricate nature of mortality trends. One viable approach to surmount the aforementioned challenges is the implementation of a Medical Board autopsy, wherein doctors from various specialties, corresponding to the clinical aetiology of death in hospitalized patients and the probable aetiology in other cases, collaboratively engage in the execution of a medico-legal autopsy.

The autopsy performed by the Medical Board is regarded as exceptionally high in quality and is esteemed by the judiciary. Such autopsies employ the most qualified personnel and resources available. Given the extensive time, administrative coordination, and effort required for the Medical Board autopsy, it becomes virtually unfeasible to manage every medico-legal case in this manner; hence, it is reserved for specific exceptional circumstances. In India, the requisition for Medical Board autopsies is primarily determined by the state and district administration, often influenced by extraneous factors, including political pressures and media sensitivity, rather than strictly objective medical or legal criteria. This arbitrariness is further exacerbated by public expectations for transparency in high-profile or custodial death cases, leading to the frequent constitution of Medical Boards even for cases that could be managed at the routine level. As a result, the motivations behind the establishment of a Medical Board for conducting medico-legal autopsies frequently lead to overburdening of the forensic infrastructure, delays in autopsy reporting, and strain on already limited forensic manpower in many parts of India. Therefore, it is imperative that the criteria governing the initiation of Medical Board autopsies be critically evaluated and made more objective, ideally through the formulation of standardized national guidelines under the aegis of central forensic and judicial authorities.

A preliminary effort in this context is to conduct an examination of the justifications for performing Medical Board autopsies in relation to the underlying manner and cause of death. Limited long-term longitudinal studies have been undertaken in this field within the nation. This examination scrutinizes the rationales for autopsies performed by Medical Boards in relation to the corresponding manner and causation of death over an extended period, with the objective of establishing a referral profile for such cases within this region and recommending possible revisions to government policy, particularly in terms of the development of guidelines for the conduction of Medical Board autopsies, thereby ensuring the equitable allocation of resources, whether human or financial, for this purpose.

## Materials and methods

The investigation carried out was arranged as a retrospective observational assessment that concentrated on records, executed at the Department of Forensic Medicine and Toxicology, Rajendra Institute of Medical Sciences (RIMS), Ranchi, Jharkhand, India. The autopsy files associated with a five-year interval, specifically between April 1, 2015, and March 31, 2020, were pinpointed, extracted, and thoroughly scrutinized to accomplish the analysis objectives.

The autopsy case documentation within the Department of Forensic Medicine and Toxicology is systematically organized in various formats. This comprehensive archive contains a diverse array of medico-legal records, including detailed findings from post-mortem examinations, police/magistrates' inquest reports, histopathological assessments of organ and tissue samples, toxicological evaluations of viscera and bodily fluids, biological evidence documentation, and additional supplementary investigative outcomes. Notably, the records included both standard medico-legal autopsies and those required by the establishment of a Medical Board, the latter representing the primary concentration of this research. The pertinent autopsy records related to Medical Board autopsies were meticulously evaluated for thoroughness, with only those that met the established criteria being selected for inclusion in the study to guarantee the integrity of the data.

The criteria for inclusion in the study were stipulated as all autopsies that required the convening of a Medical Board during the five-year period under review. Records deemed incomplete or ineligible, which posed challenges in interpretation, were excluded from the analysis. Following the stringent application of these criteria, a total of 262 autopsy records from the Medical Board were found eligible and were integrated into the final dataset for thorough analysis. A consecutive sampling approach was employed, whereby all qualifying cases within the designated timeframe were included.

To ensure systematic and coherent data collection, a comprehensive case report form (CRF) was meticulously developed prior to the initiation of data retrieval. The CRF was designed based on an extensive review of the existing literature and guidelines relevant to medico-legal autopsy documentation. It was organized into four primary sections, systematically capturing critical elements such as detailed findings from post-mortem examinations, police/magistrates' inquests, documentation of the definitive cause and manner of death, and exhaustive histopathological and toxicological assessments.

Subsequent to the preliminary completion of the data collection phase, the process of data entry commenced utilizing Microsoft Office Excel (Microsoft Corporation, Redmond, USA). The pre-analysis formatting of the dataset was conducted with great precision to ensure the standardization of variables and the minimization of errors. After finishing the data collection phase, the organized dataset went through an extensive statistical examination with the Statistical Package for the Social Sciences (IBM Corp., Armonk, USA). Descriptive statistics, encompassing frequency distributions and proportions, were produced and encapsulated in frequency tables to aptly illustrate the observed patterns.

Before the study began, essential ethical clearance was granted by the Institutional Ethics Committee (IEC) of Rajendra Institute of Medical Sciences, Ranchi (Memo no. 22), thus confirming alignment with the ethical principles applicable to research with retrospective human data. The study adhered stringently to ethical guidelines pertaining to confidentiality and anonymity. Throughout the data collection process, no identifiers related to patients or cases were employed at any juncture. All acquired information was administered with utmost confidentiality, with both physical and digital records being securely maintained in locked facilities under the oversight of the principal investigator. Upon completion of their use, the records were systematically archived to avert unauthorized access, thereby upholding the ethical and legal standards mandated for such research.

## Results

A substantial proportion of the autopsies, specifically 67.9% (178), were conducted as a consequence of custodial deaths, whereas law enforcement scenarios accounted for 12.6% (33) and prompted the subsequent highest number of autopsies (Table 1).

**Table 1:** Distribution of autopsies performed according to the reason for autopsy by a Medical Board

Reason for autopsy by Medical Board	Frequency (n=262)	Percentage (%)
Alleged medical negligence	08	3.0
Custodial death	178	67.9
Law & order situation	33	12.6
Maoist attack	11	4.2
Hooch tragedy	14	5.4
Mass suicide-homicide	12	4.6
Decomposed (apart from Custodial deaths)	5	1.9
Body highly burnt	1	0.4
Total	262	100

In a significant proportion, 62.5% (five) of the instances concerning purported medical malpractice, the medical institution failed to elucidate the cause of death. In the remaining instances, the explanations furnished included cardio-respiratory arrest; aspiration of milk during lactation, resulting in asphyxiation of the infant; and the patient experiencing a seizure episode followed by collapse (Table 2).

**Table 2:** Distribution of autopsies performed according to whether cause of death was provided by health care facility or not, in cases of medical negligence

Cause of death provided by health facility	Frequency (n=8)	Percentage (%)
Yes	03	37.5
No	05	62.5
Total	08	100

The incidence of sexual assault and homicide, accounting for 36.3% (12) and 30.3% (10) respectively, constituted the principal motivations for the execution of autopsies by the medical board in circumstances pertaining to law enforcement and public order (Table 3).

**Table 3:** Distribution of autopsies performed according to the cause of the law-and-order situation

Law & order situation	Frequency (n=33)	Percentage (%)
Homicide	08	24.3
Homicide / Re-postmortem	01	3.0
Homicide / Decomposed	01	3.0
Communal disharmony	01	3.0
Exhumation	02	6.1
Child trafficking and Gangrape	01	3.0
Rape & murder	10	30.3
Rape & murder / Burn	01	3.0
Rape & murder / Decomposed	01	3.0
Mob lynching	01	3.0
Alleged foul play	06	18.3
Total	33	100

**Figure 1:**
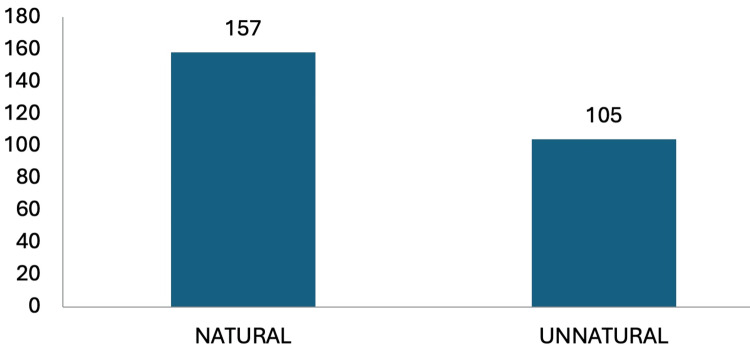
Distribution of autopsies performed according to the manner of death (n=262)

A majority, specifically 59.9% (157) of the autopsy examinations, indicated a determination of natural causes of death, while 40.1% (105) were attributed to causes deemed unnatural (Figure 1).

Lung pathologies, 19.8% (31), represented the predominant cause of natural deaths observed in the conducted autopsies, followed by cardiovascular diseases, 14.6% (23). In 25.6% (40) of the instances, the involvement of multiple organs was noted (Table 4).

**Table 4:** Distribution of natural deaths according to the cause of death

Cause of natural death	Frequency (n=157)	Percentage (%)
Heart disease	23	14.6
Multi Organ Failure	40	25.6
Kidney disease	16	10.2
Carcinoma	20	12.7
Liver disease	07	4.5
Brain disease	08	5.0
Lung disease	31	19.8
Other natural causes	12	7.6
Total	157	100

Trauma accounted for 35.2% (37) of the total cases, rendering it the predominant cause of unnatural deaths observed in the autopsies conducted, while asphyxia, comprising 31.4% (33), emerged as the second most prevalent cause of death (Table 5).

**Table 5:** Distribution of unnatural deaths according to the cause of death

Cause of unnatural death	Frequency (n=105)	Percentage (%)
Trauma	37	35.2
Asphyxia	33	31.4
Poisoning	23	21.9
Others	12	11.5
Total	105	100

Homicide, accounting for 58.1% (61) of cases, emerged as the predominant mode of unnatural deaths identified in the conducted autopsies, followed by accidental deaths at 21.9% (23) and suicides at 21% (61) (Table 6).

**Table 6:** Distribution of autopsies performed according to the manner of unnatural death

Manner of death	Frequency (n=105)	Percentage (%)
Suicide	21	20
Homicide	61	58.1
Accident	23	21.9
Total	105	100

In more than half, 57.1% (12), of the suicide cases, hanging was the method of suicide (Table 7).

**Table 7:** Distribution of autopsies performed according to the method of suicide in suicidal deaths

Method of suicide	Frequency (n=21)	Percentage (%)
Burn	01	4.8
Hanging	12	57.1
Poisoning	07	33.3
Self-inflicted head injury	01	4.8
Total	21	100

The predominant methodology of homicide involved trauma inflicted by hard and blunt objects, accounting for 26.3% (16), followed by injuries resulting from firearms, which constituted 16.4% (10) (Table 8).

**Table 8:** Distribution of autopsies performed according to the mode of homicide

Method of homicide	Frequency (n=61)	Percentage (%)
Firearm	10	16.4
Hard & blunt object	16	26.3
Hard & blunt object + Ligature strangulation	02	3.3
Hard & blunt object + Poisoning	01	1.6
Hard & blunt object + Manual Strangulation	01	1.6
Landmine blast	06	9.9
Ligature strangulation	08	13.1
Ligature strangulation + Sharp & pointed weapon	01	1.6
Metallic arrow / Sharp & pointed weapon	01	1.6
Poisoning	07	11.5
Poisoning & Manual Strangulation	03	4.9
Manual Strangulation	02	3.3
Drowning	01	1.6
Inconclusive	02	3.3
Total	61	100

A substantial proportion, 60.9% (14), of the accidental deaths were caused by poisoning (Table 9).

**Table 9:** Distribution of autopsies performed according to the cause in accidental deaths

Mode of injury	Frequency (n=23)	Percentage (%)
Burn	01	4.3
Drowning	01	4.3
Electrocution	02	8.7
Hard & blunt substance	04	17.5
Poisoning	14	60.9
Asphyxia after seizure	01	4.3
Total	23	100

## Discussion

The findings from the present study, conducted on medico-legal autopsies, offer significant insights into patterns of death under exceptional circumstances. During the study period, a total of 15,683 medicolegal autopsies were conducted, out of which 262 cases (1.7%) warranted autopsies by Medical Boards, highlighting the selective and exceptional nature of Medical Board involvement.

A large majority (67.9%) of the Medical Board autopsies were conducted due to custodial deaths, reflecting the heightened legal and public scrutiny surrounding deaths occurring within institutional custody, such as jails and police lock-ups. This is consistent with patterns seen nationally, where custodial deaths often necessitate independent, higher-level scrutiny to ensure transparency and accountability. Law-and-order situations (12.6%) formed the next major category requiring Medical Board autopsies, particularly in sensitive cases such as rape and murder (36.3%), homicides (30.3%), and deaths following incidents such as mob lynching, communal violence, and gang rape. This underscores the crucial role of Medical Boards in politically or socially sensitive cases where public trust must be maintained. Interestingly, within the subset of law-and-order-related autopsies, a significant proportion of bodies were either charred or decomposed, posing considerable challenges for forensic evaluation. These findings emphasize the complex nature of forensic examination in such conditions, requiring a higher expertise level provided by Medical Boards.

In regard to the cause of death, a substantial proportion (59.9%) of the autopsied cases were classified as resulting from natural causes, which is consistent with outcomes from other investigations conducted in India [11-14]. The dominance of pulmonary diseases (19.8%) as the primary contributor to natural deaths within our cohort, followed by cardiac ailments (14.6%), has also been documented by other researchers [15-16]. However, this observation contrasts with numerous studies from Western countries and urban Indian locales where cardiovascular diseases (notably ischemic heart disease) are prevalent [17-18]. This discrepancy may be attributed to regional influences, as Ranchi and its adjacent areas exhibit a significant incidence of respiratory diseases, particularly among economically disadvantaged groups, miners, and rural workers, due to factors including poverty, air pollution, occupational risks, and tobacco use. Additionally, the high incidence of custodial deaths within the study demographic could have influenced this outcome, given that individuals in custody frequently originate from vulnerable and marginalized communities, who are at an elevated risk of undiagnosed or untreated pulmonary conditions resulting from chronic neglect of healthcare services. Extended detention in overcrowded and inadequately ventilated facilities further intensifies the deterioration of respiratory illnesses, thereby amplifying their role in deaths within custodial environments. Such respiratory ailments are less common in more urbanized and industrialized settings where lifestyle-related diseases like ischemic heart disease predominate. Moreover, the observation that multiple organ involvement was documented in 25.6% of cases indicates a considerable prevalence of multisystem diseases, likely indicative of postponed health-seeking behaviours, insufficient healthcare access, or chronic comorbidities prevalent within the population served by the institution.

Among unnatural deaths (40.1%), homicides (49.5%) significantly outnumbered accidents (21.9%) and suicides (17.2%), contrasting with several other studies where suicides predominate among unnatural deaths [15,19]. This pattern can be attributed to the selective nature of Medical Board autopsies in our context, routine suicides and accidental deaths are often managed by individual forensic experts, whereas deaths suspected to have legal, political, or communal implications, such as homicides, are escalated to Medical Boards.

Regarding suicide methods, hanging (57.1%) was the most frequent, followed by poisoning (33.3%), which aligns with the National Crime Records Bureau (NCRB) Accidental Deaths and Suicides in India (ADSI) 2019 report [20] and various Indian studies [21-22]. Nevertheless, some regions have documented poisoning as the most common method [23-24], possibly due to easier access to pesticides in agricultural belts. The predominance of hanging in our findings might be explained by urbanization trends and greater awareness of lethal methods among the population.

In the domain of homicides, the predominance of blunt force trauma (34.9%) is congruent with multiple other Indian studies [25-26], suggesting that blunt instruments remain the most commonly used weapons in interpersonal violence, likely due to their widespread availability. However, some studies have reported sharp weapon injuries as more prevalent [27], which may reflect regional cultural differences, weapon availability, or modus operandi preferences in different parts of India.

In accidental deaths, poisoning (60.9%), notably from hooch tragedies, was the leading cause, diverging from National Crime Records Bureau (NCRB) Accidental Deaths and Suicides in India (ADSI) 2019 report where road traffic accidents are the major contributors [20], which also is in conformity with works done in other countries [28,29]. This discrepancy likely arises because Medical Board autopsies are selectively ordered for mass casualties (like hooch tragedies) or controversial deaths, whereas routine traffic accident deaths often proceed with standard autopsies. Additionally, in areas like Jharkhand, illicit alcohol consumption remains a critical public health issue, occasionally leading to mass poisoning events requiring higher scrutiny.

The unique findings of deaths from landmine blasts (Maoist insurgencies) also highlight the regional conflict-specific violence seen in eastern India, differentiating our study population from other areas less affected by insurgency-related violence.

Thus, while several findings in this study resonate with national and international trends, many variations can be attributed to region-specific health profiles, socioeconomic conditions, public health challenges, patterns of violence, and the selective nature of cases referred to Medical Boards. Understanding these differences is crucial for tailoring forensic services, public health interventions, and policy formulation to regional realities.

Limitations

The results of this monocentric investigation may hold distinct significance for the Jharkhand region, thereby constraining their applicability to other geographical locales or nations, where regional epidemiological trends, healthcare systems, and socio-political contexts vary considerably. A notable limitation relates to the intrinsic selection bias linked to the cases undergoing Medical Board autopsies. The determination to establish a Medical Board is frequently shaped by external administrative, legal, or political factors, rather than being founded solely on objective medical criteria, which may distort the case profile towards more contentious or high-profile deaths, such as custodial deaths, alleged police misconduct, or politically sensitive events. This selective inclusion of cases may inadvertently amplify specific manners or causes of death (e.g., custodial deaths) while diminishing the representation of others that typically do not raise administrative alarm. 

Additionally, inadequate and inconsistent records from healthcare institutions presented a significant challenge, especially in instances of alleged medical negligence, where the primary treating facilities often neglected to provide a definitive cause of death or sufficient clinical details. This deficiency in information could have undermined the precision of final autopsy determinations and restricted the thoroughness of post-mortem medico-legal evaluations. Furthermore, the retrospective design of the study inherently limited the capacity to verify or augment missing or unclear data points. 

Collectively, these limitations highlight the imperative for more extensive, multicentric investigations, preferably orchestrated by national bodies such as the Bureau of Police Research and Development (BPR&D) under the Ministry of Home Affairs, Government of India, integrating multidisciplinary contributions from forensic specialists, district governance, law enforcement, and public health authorities to more effectively illuminate the systemic factors propelling the rising demand for Medical Board autopsies and to devise evidence-based policy interventions.

## Conclusions

Autopsies performed by Medical Boards offer significant advantages in fostering a broader perspective for ensuring impartiality, are subjected to more rigorous examination, and have garnered greater confidence from the judiciary compared to autopsies not conducted by Medical Boards. The additional temporal, material, financial, and logistical complexities that accompany Medical Board autopsies do not render them practically feasible in every case across all autopsy centres within our nation. The typology of cases addressed by boards, the methodologies employed in conducting such autopsies, the duration required to arrive at a conclusive opinion, and ultimately the documentation and reports submitted in legal proceedings vary considerably throughout the country. The supplementary temporal, material, financial, and logistical challenges inherent to Board autopsies impede their practical feasibility across all circumstances at diverse autopsy facilities within our nation.

Consequently, it is essential that detailed guidelines are formulated concerning the categorization of cases designated for Medical Board autopsy, the structural framework of the board to be constituted, and the procedural protocols to be observed during such autopsies in order to attain a definitive conclusion, even in scenarios characterized by insufficient infrastructure. This is vital to ensure that the execution of autopsy by Medical Boards maintains the trust and reliance of legal authorities and the judiciary, without imposing an excessive strain on the limited resources of the Forensic Medicine and Toxicology departments. Furthermore, undertaking comprehensive research on the trends of medicolegal autopsy cases conducted by Medical Boards across various regions of the nation will promote the formulation of a protocol to more effectively tackle the practical challenges faced in these cases, particularly in inadequately equipped facilities throughout the country.
